# Automatic decomposition of electrophysiological data into distinct nonsinusoidal oscillatory modes

**DOI:** 10.1152/jn.00315.2021

**Published:** 2021-10-06

**Authors:** Marco S. Fabus, Andrew J. Quinn, Catherine E. Warnaby, Mark W. Woolrich

**Affiliations:** ^1^Oxford Centre for Functional MRI of the Brain, Nuffield Department of Clinical Neurosciences, University of Oxford, Oxford, United Kingdom; ^2^Wellcome Centre for Integrative Neuroscience, University of Oxford, Oxford, United Kingdom; ^3^Oxford Centre for Human Brain Activity, Department of Psychiatry, University of Oxford, Oxford, United Kingdom

**Keywords:** EMD, neural oscillations, nonsinusoidal

## Abstract

Neurophysiological signals are often noisy, nonsinusoidal, and consist of transient bursts. Extraction and analysis of oscillatory features (such as waveform shape and cross-frequency coupling) in such data sets remains difficult. This limits our understanding of brain dynamics and its functional importance. Here, we develop iterated masking empirical mode decomposition (itEMD), a method designed to decompose noisy and transient single-channel data into relevant oscillatory modes in a flexible, fully data-driven way without the need for manual tuning. Based on empirical mode decomposition (EMD), this technique can extract single-cycle waveform dynamics through phase-aligned instantaneous frequency. We test our method by extensive simulations across different noise, sparsity, and nonsinusoidality conditions. We find itEMD significantly improves the separation of data into distinct nonsinusoidal oscillatory components and robustly reproduces waveform shape across a wide range of relevant parameters. We further validate the technique on multimodal, multispecies electrophysiological data. Our itEMD extracts known rat hippocampal θ waveform asymmetry and identifies subject-specific human occipital α without any prior assumptions about the frequencies contained in the signal. Notably, it does so with significantly less mode mixing compared with existing EMD-based methods. By reducing mode mixing and simplifying interpretation of EMD results, itEMD will enable new analyses into functional roles of neural signals in behavior and disease.

**NEW & NOTEWORTHY** We introduce a novel, data-driven method to identify oscillations in neural recordings. This approach is based on empirical mode decomposition and reduces mixing of components, one of its main problems. The technique is validated and compared with existing methods using simulations and real data. We show our method better extracts oscillations and their properties in highly noisy and nonsinusoidal datasets.

## Graphical abstract

**Figure:**
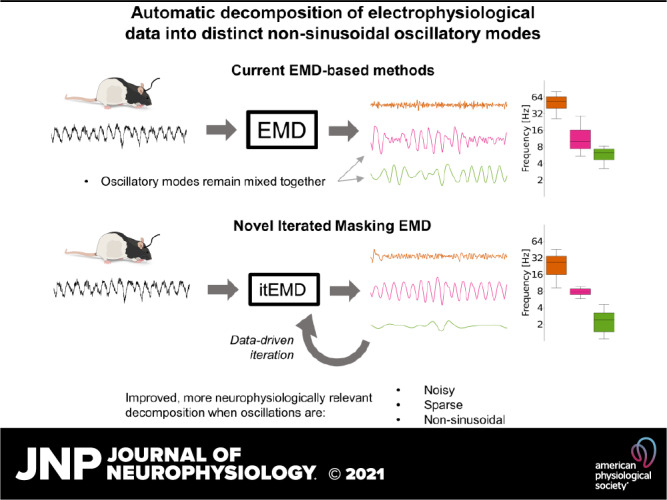

## INTRODUCTION

The synchronized activity of neuronal populations can be observed in dynamic oscillations recorded in electrophysiology (1, 2). These oscillations are often visible in raw data traces but are challenging to isolate in an objective, data-driven manner. Methods for signal isolation must contend with signals being obscured by noise or by simultaneous oscillations at different frequencies. Neuronal oscillations are often nonsinusoidal and change over time, which leads to ambiguities in standard analyses based on the Fourier transform (3, 4). These dynamic and nonsinusoidal features are of growing importance in electrophysiological research but remain difficult to analyze using existing methods (1, 5–9). As such, there is a pressing need for data-driven methods that can isolate oscillations from noisy time-series while preserving their nonsinusoidal features.

Empirical mode decomposition (EMD; 10) is able to provide a different perspective on analyzing transient oscillations. It offers a radically different approach to signal separation based on a flexible, local, and data-driven decomposition with weaker assumptions about stationarity and linearity of the signal. Single-channel data is decomposed by a sifting process into intrinsic mode functions (IMFs) based on finding successively slower extrema. Unlike Fourier or wavelet methods, EMD does not a priori assume the shape of its functions. It is, therefore, believed that IMFs can capture nonsinusoidal oscillations and may better reflect the underlying processes in physical and physiological signals (3, 10, 11). This can especially aid analyses sensitive to waveform shape, such as calculations of phase and cross-frequency coupling (4, 12).

The original EMD algorithm can in theory produce arbitrarily shaped IMFs, but in noisy neural signals it struggles with signal intermittency and high nonsinusoidality. In the presence of transient oscillatory bursts, the sifting process may detect extrema on different time scales at different times. This is referred to as mode mixing. It presents a major challenge in analysis and interpretation of IMFs (13, 14). This is especially the case in analysis of brain signals, where transient states are common and have functional significance (5, 15–17). Furthermore, in the presence of pure Gaussian fractional noise, EMD has been shown to act as a dyadic filter bank (18, 19). This means that for highly noisy signals, EMD tends to produce IMFs with fixed bandwidths rather than adapting to capture signals present in the data, further complicating the analysis.

Various improvements to the sifting process have been proposed to make EMD more applicable to real-world data (20–27). A unifying characteristic of the existing approaches is to inject a secondary signal into the data to alter the extrema distribution and overcome mode mixing. Noise-assisted methods, as exemplified by ensemble EMD (EEMD) (20, 22), use white noise as the injected signal. This reduces mode mixing due to signal intermittency. However, the use of noise can limit IMF bandwidth, possibly making mode mixing worse. Masking methods inject sinusoids into the data before sifting (21, 24). With a suitable mask, this technique can recover nonsinusoidal waveforms and/or intermittent bursts in the presence of noise. However, the frequency of masking signals that should be used is often not known a priori. Mask optimization can become an arduous manual process, prohibiting generalizability and introducing uncertainty on analysis outcomes. This is exacerbated by the presence of high noise and nonsinusoidal signals near dyadic boundaries, where a small change in the masking signal frequency may dramatically alter the quality of the resulting IMFs. Mask frequency selection can be done semiautomatically by choosing an initial frequency based on the number of zero-crossings in the first IMF and dividing this successively by two for later IMFs (21). If the approximate frequency content of the signal is known, then mask frequencies may be directly selected to isolate the specific components of interest (11). Though effective, the semiautomatic method is relatively inflexible, and the direct specification method can be manually intensive to validate. Finally, multivariate EMD is also a subject of active research (28, 29). The extension of EMD to multichannel data is not trivial as interpolating extremal envelopes becomes computationally expensive in higher dimensions and additional methods are needed, such as only sifting along most important directions. Alternatively, pseudo-multivariate EMD can be computed by simply performing EMD on each channel separately and checking cross-channel mode correspondence afterward, for example, by comparing their frequency content.

In this paper, we introduce iterated masking EMD (itEMD), a novel sifting technique that builds on the masking method. This method retains all the advantages of using a masking signal while being more generalizable and automated. We compare itEMD with existing methods using simulations and multispecies, multimodal experimental data, and discuss its range of applicability and limitations.

In simulations, we have focused on the three areas important to the analysis of neural signals, as aforementioned: noise, sparsity, and waveform shape distortion. All three of these cause major issues for EMD and limit its applicability to neurophysiology. Here, we show that itEMD performs significantly better than existing methods in cases with highly noisy and strongly nonsinusoidal signals, where our technique significantly reduces mode mixing and accurately extracts oscillations and their shape. We further validate the technique by analyzing oscillations in rat local field potential (LFP) data and human magnetoencephalography (MEG) recordings. We show that itEMD reproduces the well-known hippocampal θ waveform shape better than existing techniques and successfully reconstructs occipital α peak frequency with no a priori information about mask frequencies. Furthermore, itEMD significantly reduced mode mixing in both datasets studied.

## MATERIALS AND METHODS

### Data and Code Availability Statement

All figures and analysis in this paper can be freely replicated with Python code available at https://gitlab.com/marcoFabus/fabus2021_itemd. Hippocampal LFP data are available from the CRCNS platform (https://crcns.org/data-sets/hc/hc-3) and human MEG data are available from the Cam-CAN archive (https://camcan-archive.mrc-cbu.cam.ac.uk/dataaccess/) (30, 31). Analyses were carried out in Python 3.9.4, building on the open-source EMD package (v0.4.0), available with tutorials at https://emd.readthedocs.io (32). Underlying dependency packages were numpy (33), scipy (34), and statsmodels (35) for computation and matplotlib (36) for visualization.

### EMD Algorithms

Empirical mode decomposition decomposes a signal *x*(*t*) into a finite number of intrinsic mode functions (IMFs) *c_i_* with a sifting algorithm (10). The IMFs are constructed to have locally symmetric upper and lower envelopes with no peaks below zero or troughs above zero. A smooth signal with these features is well-behaved during instantaneous frequency analysis, allowing for a full description of nonsinusoidal waveform shape (11).

Ensemble EMD (20) is typical of a class of noise-assisted sifting methods. An ensemble of *n* sift processes is created, each with different white noise injected. The final IMFs are computed as the average across this ensemble. The goal is to exhaust all possible sifting solutions, leaving only persistent real signals. However, due to a finite size of the ensemble, IMFs may contain unwanted residual noise unless further improvements are introduced (25, 26). Furthermore, due to the stochastic nature of white noise, signals of interest might shift between modes across the ensemble, leading to some mode mixing in the final result. Finally, the use of noise reinforces the dyadic filtering behavior of EMD. This means any signal near dyadic boundaries is likely to be split between modes. This effect is especially pronounced for nonsinusoidal signals which change in instantaneous frequency, making waveform shape analysis difficult as they become smeared over multiple IMFs.

Masked EMD (21) works by injecting a masking signal *s_i_*(*t*) into signal *x*(*t*) before sifting. This reduces mode mixing by making the sift ignore signal content slower than the frequency of the masking signal. The masking signal is introduced uniformly across *n_p_* phases at each step to further minimize mode mixing (22). The IMFs *c_i_* are thus calculated with the following algorithm:

1. Construct a masking signal *s_i_*(*t*).
2. Perform EMD on *x_k_* = *x*(*t*) + *s_i_*_,_*_k_*(*t* + *φ_k_*), where *φ_k_* = 2*π*(*k* − 1)/*n_p_*, obtaining IMFs *c_i_*_,_*_k_*(*t*).
3. Compute the final IMF as *c_i_*(*t*) = 1/*n_p_* ∑*c_i_*_,_*_k_*.
4. Compute the residue *r_i_*(*t*) = *x*(*t*) – *c_i_*(*t*).
5. Set *x*(*t*) = *r_i_*(*t*) and repeat 1–4 with the next masking signal to extract the next IMF.

**Figure 1.**
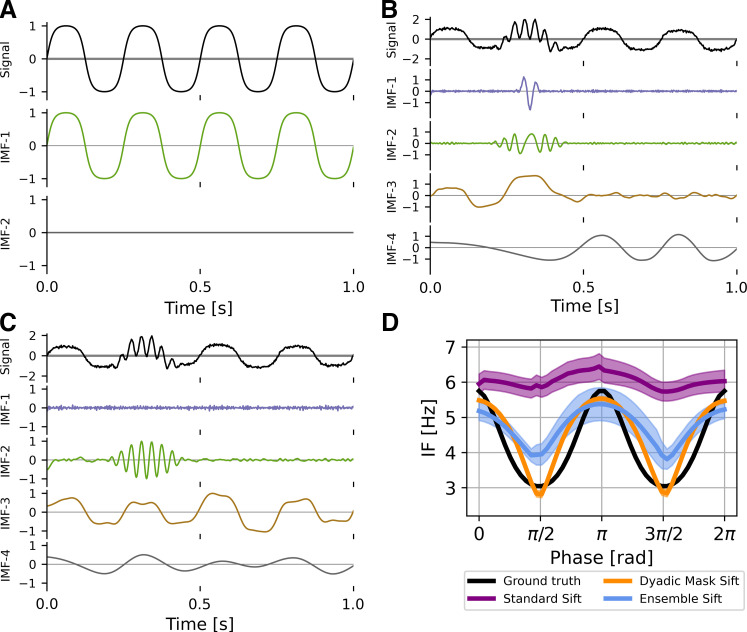
Limitations of empirical mode decomposition (EMD). *A*: standard EMD sifting applied on a pure 4 Hz iterated sine function. With no noise, EMD can accurately identify an intrinsic mode function (IMF) that represents the nonsinusoidal signal. *B*: in the presence of white noise and a 30-Hz burst (arrow), standard EMD shows heavy mode mixing. *C*: EMD with an appropriate dyadic mask sift will recover most of the iterated sine (IMF-3) and the intermittent burst (IMF-2) signals. *D*: masked EMD and ensemble EMD can better reconstruct nonsinusoidal wave shape in signal with low noise unlike standard EMD. The figure shows the phase-aligned instantaneous frequency calculated from 100 runs of *B* and *C*. Means ± standard error (shaded) shown.

This technique permits analysis with intermittent bursts and nonsinusoidal oscillations (Fig. 1). EMD is locally adaptive, and as such fast bursts get mixed with slower activity when bursts are not present. With a mask, any signal content with frequencies much lower than the masking frequency will be ignored by the sift in that iteration and is replaced by the mask. The mask is finally removed allowing us to recover intermittent activity correctly. In the presence of noise, EMD also acts as a dyadic filter (18, 19). This means nonsinusoidal oscillations are often split across multiple IMFs. With a suitable mask, the bandwidth of modes can be adapted and more of the waveform shape recovered.

The choice of masking signals remains an area of active research. The original paper by Deering and Kaiser (21) suggested the first mask frequency to be the energy-weighted mean of instantaneous frequency obtained from the first IMF found by ordinary EMD, with subsequent mask frequencies chosen as *f*_0_ divided by powers of 2 to account for the dyadic nature of EMD. Other approaches have included computing the mask from zero crossings of the first IMF of a standard sift and purely dyadic masks (24). However, the choice of optimal masks remains a manual process in many cases. This requires experience and may introduce subjective bias (11, 21, 27, 37).

### Iterated Masking EMD

As seen earlier, noise-assisted and masking approaches to EMD sifting improve mode mixing in some cases, but mode mixing may still be present to complicate further analysis. Mask choice in noisy datasets is complicated, especially with signal frequencies near dyadic boundaries. Iterated masking solves this problem by finding and using an adaptive, data-driven mask.

In science, it is common to rely on intuition to guide study of complex dynamical systems (38). Consider then a simple example where there is a signal burst *x*(*t*) with some base frequency *f*_sig_ and possible deviation around it due to nonsinusoidality. Take as a start the masked EMD process with a single mask of frequency $f_{\text{mask}}^{\left(0\right)}$. A good choice of frequency would be near *f*_mask_ = *f*_sig_, as this would extract most of *x*(*t*) into one IMF, resulting in noise reduction and allowing for a simple IMF interpretation. This is because adding a mask at *f*_mask_ = *f*_sig_ forces the IMF to ignore any spectral content below ∼0.67 × *f*_mask_ (27).

In real data however, *f*_sig_ is often unknown. Assume then $f_{\text{mask}}^{\left(0\right)}$ is chosen with little to no knowledge of the system frequency *f*_sig_. After applying masked EMD, the resulting IMF will contain a part of the burst with some noise or other signal mixed in. However, its instantaneous frequency will be *f*_sig_ for sections of the IMF attributable to the signal. Assuming signal amplitude is distinguishable from noise in this IMF, the amplitude-weighted instantaneous frequency mean (AW-IFM) will be closer to the desired *f*_sig_ than $f_{\text{mask}}^{\left(0\right)}$. Thus, if we use this AW-IFM as the masking frequency for the next iteration $f_{\text{mask}}^{\left(1\right)}$, the resulting mask sift IMF will be even closer to the optimal IMF. This is the case if both $f_{\text{mask}}^{\left(0\right)}$ is greater and smaller than *f*_sig_, as both lead to mode mixing. Following this reasoning, the natural equilibrium of this iteration process is when *f*_mask_ = *f*_sig_, and we can apply this approach to a signal consisting of multiple signal frequencies and noise. This leads us to the following algorithm:

1. Choose an initial set of mask frequencies *m* = {*f*_0_}.
2. Perform masked EMD to obtain IMFs.
3. Find the instantaneous frequency (IF) for each IMF using the Hilbert transform.
4. Compute the amplitude-weighted average of each IMF’s IF and set *m_i_* = AW-IFM.
5. Repeat 2–4 until a stopping criterion ∑ is reached.

**Figure 2.**
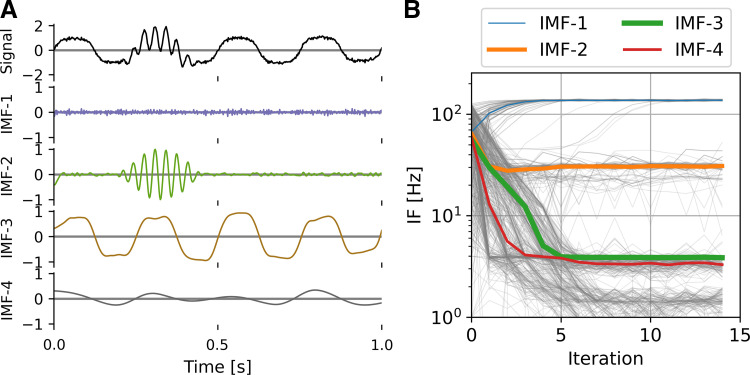
Iterated masking empirical mode decomposition (EMD) (itEMD) on simulated data. *A*: example endpoint of itEMD, i.e. intrinsic mode functions (IMFs) from last of 15 iterations on the same signal as in Fig. 1*B*. *B*: itEMD convergence to equilibrium starting with a mask drawn randomly from a uniform distribution of 1–128 Hz. Thin gray lines are all 100 individual runs, colored lines are median trajectories with line thickness scaled to maximum value of that IMF. itEMD converges rapidly, adapts mask frequencies to the signal content, and correctly finds both the nonsinusoidal 4 Hz oscillation and intermittent 30 Hz burst with no prior knowledge about the frequencies contained in the signal.

Here, the stopping criterion was chosen such that the relative difference between current and previous mask frequencies is small, i.e., (*m_i_* – *m_i_*_−1_)/−*m_i_*_−1_ < ∑. Instantaneous frequency averaging was weighted by the square of instantaneous amplitude for a given IMF, i.e., by instantaneous power. Mask frequencies were initialized by the dyadic masking technique, though it was found that itEMD is not sensitive to mask changes and can rapidly identify correct IMFs even with a random initial mask (Fig. 2). Due to rapid convergence (<10 iterations in most cases), itEMD is computationally comparable with existing techniques including ensemble EMD and uniform phase EMD, each of which requires repeated sifting (22). More formally, the computational complexity of itEMD is *T* = 41*n*_iter_ × *n*_s_ × *n*_p_ × *n* log_2_(*n*) for *n*_iter_ iterations, *n*_s_ sifting steps, *n*_p_ mask phases, and data length *n*.

### Simulations

We ran simulations to compare the performance of itEMD to existing sifting methods, namely ensemble EMD (EEMD) (20) and masked EMD (21). Simulations were performed along three dimensions that are important to analysis of neural signals: noise, sparsity, and waveform shape distortion (nonsinusoidality). These were chosen as they are all common features of neurophysiological data which cause issues for extracting neural oscillations. In standard EMD, they result in mode mixing and prohibit accurate representation of waveform shape and robust interpretation of identified modes.

All noise and frequency distortion simulations were 10-s long and sampled at 512 Hz with signal amplitude normalized to 1. In each simulation, IMFs were computed using three different methods that are used to address mode mixing: dyadic mask sift, ensemble sift, and our novel iterated masking EMD (itEMD). Dyadic mask sift utilized a single set of masking frequencies. The first was computed from zero-crossings of first IMF obtained by a standard sift and subsequent masking frequencies were divided by powers of 2. Masks were applied with four phases uniformly spread across 0 to 2*π* following Wang et al. (22). Ensemble sift was run with four noise realizations and ensemble noise standard deviation of 0.2. The novel itEMD was run on top of the masked EMD implementation with a stopping criterion ∑ = 0.1 and maximum number of iterations *n*_max_ = 15. In all simulations, number of IMFs was limited to 6 and the sifting threshold was 10^−8^. After finding IMFs, individual cycles were found from jumps in the instantaneous phase found by the amplitude-normalized Hilbert transform. Each set of simulations (noise, distortion, sparsity) was repeated *n* = 100 times with the means ± standard error results presented.

Waveform shape was quantified by computing the average phase-aligned instantaneous frequency (IF) across cycles (11). IF measures how an oscillation speeds up or slows down within a cycle. It is computed as the time derivative of the instantaneous phase. IF was phase-aligned to correct for differences in timing and duration between cycles and allow for comparisons at each phase point. It can intuitively be understood as fitting a sinusoid with frequency that of the instantaneous frequency at each time point, capturing shape deviations away from a sinusoid with a constant frequency (39, 40). Within-cycle IF variability is thus a measure of how nonsinusoidal each cycle is.

Performance of each method was assessed by two methods. The first was finding Pearson correlation between reconstructed phase-aligned instantaneous frequency (proxy for waveform shape) and its ground truth. The second was computing the pseudo-mode splitting index (PMSI) introduced by Wang et al. (22). PMSI estimates the degree of mode mixing between two adjacent IMFs by computing the normalized dot product between them:

(*1*) $${\text{PMSI}}_{i,i+1}=\text{max}\left(\frac{\vec{c_{i}}\cdot \vec{c_{i+1}}}{{\left|c_{i}\right|}^{2}+{\left|c_{i+1}\right|}^{2}},0\right).$$

Orthogonal, well-separated modes with no mode mixing thus have PMSI = 0. Fully split modes have PMSI = 0.5. This index was chosen as it can be applied to both simulated and real data and is easy to interpret. For simulations with a known ground truth, we took the IMF of interest as the one with mean instantaneous frequency closest to that of the ground truth and calculated PMSI as the sum of PMSIs with the above and below IMF.

#### Noise simulations.

For analyzing noise-dependent properties, white noise was created using the numpy.random.normal Python module with zero mean and standard deviation *σ* (also equal to its root-mean-square, RMS). White noise was chosen because performance results tested on it are independent of signal frequency. This is because white noise has equal power throughout the frequency spectrum. For simulations of neurophysiological data, signals with brown noise were also considered (see Supplemental material; all Supplemental material is available at https://doi.org/10.6084/m9.figshare.16680901.v1). For this set of simulations, white noise RMS *σ* was varied between *σ* = 0.05 and *σ* = 3 in 100 uniformly spaced steps. Waveform shape distortion was held constant at frequency distortion (FD) = 68% (see *Waveform shape distortion simulations*).

#### Waveform shape distortion simulations.

For analyzing waveform shape, signal was simulated as an iterated sine function, i.e., sin{sin[…sin(2 × *π* × *f*_0_ × *x*)]} iterated *n*_sin_ times with *f*_0_ = 4 Hz. This function was chosen because *1*) it is easy to manipulate its nonsinusoidal distortion by increasing *n*_sin_, *2*) it is well-understood analytically (41), *3*) it has been used before in context of EEG time-frequency analysis (42), and *4*) it has a well-behaved instantaneous frequency by satisfying conditions outlined by Huang et al. (10). It also qualitatively captures parts of waveform shape of the sensorimotor μ oscillation and slow oscillations in depth EEG recordings by its “flat top” structure (7, 43). The base frequency of 4 Hz was chosen as it is physiologically plausible in the δ range and was near a Nyquist boundary, where current EMD sifting methods may have issues. Its nonsinusoidality was captured by a frequency distortion metric FD defined by

(*2*) $$\text{FD}=\frac{\text{max}\left(\text{IF}\right)-\text{min}\left(\text{IF}\right)}{f_{0}}\times 100\%.$$

A signal with FD = 0% is a pure sinusoid and FD = 100% indicates a waveform with IF range equal to that of the original frequency, i.e., 4 ± 2 Hz. An example waveform can be seen in Fig. 1 (FD = 68%). In this set of simulations, frequency distortion was varied between FD= 18% and 101% by repeating simulations with iterated sine order varying from *n*_sin_ = 1 to *n*_sin_ = 18. White noise RMS was held constant at *σ* = 1.

#### Signal intermittency simulations.

For analyzing effects of signal intermittency on itEMD performance, we simulated bursts of different length in a 25-s segment of data. Sparsity was measured as the number of individual oscillations in the burst. The number of cycles in the burst was varied from 5 to 95 in 100 steps. Noise RMS was kept constant at *σ*_noise_ = 1 and distortion at FD = 68% (8th-order iterated sine).

Statistical testing was done using one-sided Welch’s *t* test corrected for multiple comparisons using Bonferroni’s method unless otherwise specified (44).

### Experimental Data

#### Rat local field potential data.

To validate our method with well-described hippocampal θ oscillations, we used a publicly available dataset of Long–Evans rats (45, 46). The full 1,000 s local field potentials (LFP) recording from rat EC-013 sampled at 1,250 Hz was used for analysis. The electrode analyzed was implanted in the hippocampal region CA1. EMD cycle analysis was the same as during simulations. In short, three types of sifting methods were compared: dyadic masking sift with zero-crossing initialization, ensemble sift, and the novel itEMD. The recording was split into 20 segments of 50 s duration before sifting. For itEMD (as in simulations), the stopping criterion was set at ∑ = 0.1, the maximum number of iterations was *n*_max_ = 15, the mask was weighted by squared instantaneous amplitude, and the iteration process was initialized by the zero-crossing dyadic mask result. Instantaneous phase, frequency, and amplitude were computed from the IMFs using the amplitude-normalized Hilbert transform with an instantaneous phase smoothing window of *n* = 5 timepoints. The θ IMF was chosen as that whose average instantaneous frequency was closest to the Fourier spectral θ peak estimated using Welch’s method (peak in 4–8 Hz, function scipy.signal.welch, 8 s segment length/0.125 Hz resolution). Cycles were computed from jumps in the wrapped instantaneous phase. To discard noisy cycles, only cycles with monotonic instantaneous phase, instantaneous amplitude above the 50th percentile, and instantaneous frequency below 16 Hz were used for further analysis. Cycles were phase-aligned with *n* = 48 phase points and the shape was represented by the mean of the phase-aligned instantaneous frequency. To compare mode mixing, the PMSI (*Eq. 1*) was also computed as the sum of PMSIs of the θ IMF with the IMF above and below it in frequency.

Finally, we also computed the Wavelet transform of the LFP data for comparison with the Hilbert–Huang transform (HHT). This was done using the scipy.signal.cwt function with the Complex Morlet wavelet with *ω*_0_ = 4 and *n* = 100 frequency points between 1 Hz and 64 Hz as the widths. HHT was computed using the emd.spectra.hilberthuang function in the same frequency range with a Gaussian image filter from scipy.ndimage with *σ* = 0.5 applied for visualization purposes.

#### Human magnetoencephalography data.

Ten resting-state MEG recordings were randomly chosen from the CamCAN project (https://www.cam-can.org/) (30, 31). The participants were randomly chosen from the project (mean age 43.5 yr, range 18–79, 6 females). The maxfilter processed data were downloaded from the server and converted into SPM12 format for further analysis using the OHBA Software Library (OSL; https://ohba-analysis.github.io/osl-docs/). Each dataset was down-sampled to 400 Hz and bandpass filtered between 0.1 and 125 Hz. Two notch filters were applied at 48–52 Hz and 98–102 Hz to attenuate line noise. Physiological artifacts were removed from the data using independent components analysis. Sixty-two components were computed from the sensor space data and artifactual components identified by correlation with EOG and ECG recordings. Any independent component with a correlation greater 0.35 with either the EOG or ECG was considered artifactual and removed from the analysis. This resulted in two to four components removed from each dataset. EMD analyses proceeded with the cleaned MEG data from a single gradiometer MEG2112 over midline occipital cortex. Each recording was ∼10 min (median 565 s, range 562–656 s). The power spectrum of the whole recording was estimated using Welch’s method (function scipy.signal.welch, 8 s segment length/0.125 Hz resolution). The frequency of the spectral α peak was then extracted in the 8–12 Hz range as a local maximum (function scipy.signal.find_peaks). For itEMD analysis, each recording was segmented into 10 parts of the same length (median segment length 56.2 s). EMD was performed with the mask sift, ensemble sift, and itEMD. Sift parameters were identical to those used for the rat LFP analysis (see *Rat local field potential data*). The IMF representing α oscillations was chosen as the one whose mean instantaneous frequency was closest to the α peak frequency. Subjects were excluded if no α peak was present (one subject). After extraction of cycles from the Hilbert-computed instantaneous phase jumps, only those with instantaneous frequency between 7 and 14 Hz and instantaneous amplitude above the 50th percentile were kept. For further analysis, cycles were phase-aligned to *n* = 48 uniformly spaced phase points between 0 and 2π and the mean across cycles was computed for each subject. To evaluate mode mixing, the PMSI for each sifting method was also calculated.

## RESULTS

### Simulations

Iterated masking sift (itEMD) rapidly converged on signal in the presence of noise and intermittency. An initial 10-s data segment with a 30-Hz transient burst, a 4 Hz nonsinusoidal oscillation, and low white noise was first simulated (Fig. 2). The iteration process was started with a set of six random masks drawn uniformly from 1 to 128 Hz. Despite this initial complete lack of knowledge about the signal, itEMD correctly recovered the nonsinusoidal waveform and the β-frequency burst. The iteration process converged with noise in IMF-1, the 30-Hz β burst in IMF-2, and nonsinusoidal 4 Hz signal in IMF-3. Subsequently, convergence was determined automatically. The convergence criterion was set to the mask stabilizing within 10% between iterations with a maximum number of iterations of 15 (see materials and methods). Further simulations were initialized with the zero-crossing masked sift results for faster convergence. All simulations were repeated with *n* = 100 different noise realizations.

#### Influence of noise.

**Figure 3.**
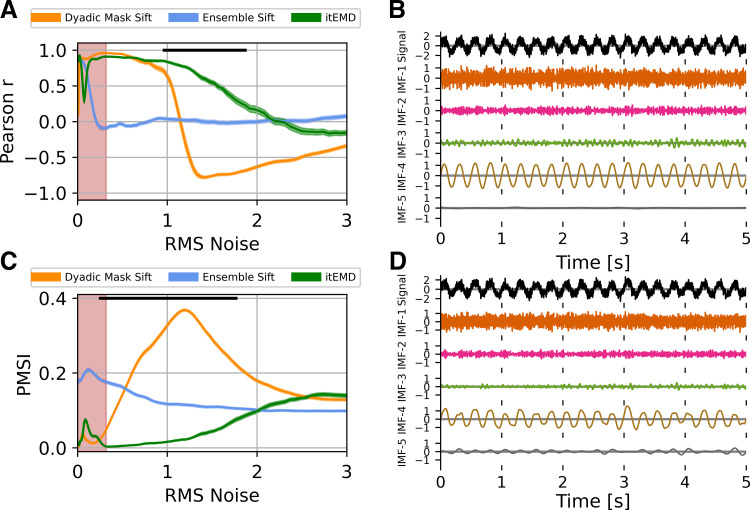
Influence of noise on empirical mode decomposition (EMD) performance on simulated data. *A*: Pearson correlation coefficient between reconstructed and ground truth instantaneous frequency against increasing white noise amplitude. *B*: example 5 s of iterated masking EMD (itEMD) sift results for *σ*_noise_ = 0.5, frequency distortion (FD) = 68%. Iterated sine function is captured by IMF-4. *C*: pseudo-mode splitting index (PMSI) against white noise amplitude, with higher PMSI values indicating higher mode mixing. Means ± standard error across *n* = 100 noise realizations shown. Black line indicates regions where itEMD performs significantly better than the best of the other techniques with *P* < 0.01 (multiple comparisons Bonferroni corrected). Red shaded region shows noise levels where itEMD reached maximum number of iterations in >20% of noise realizations. The novel itEMD performs significantly better for highly noisy data in the region between *σ*_noise_ ≈ 1 and *σ*_noise_ ≈ 2 with reduced mode mixing and accurate waveshape reconstruction. *D*: example dyadic mask sift results for *σ*_noise_ = 0.5, FD = 68%. Intrinsic mode function (IMF)-4 shows significantly more mode splitting than itEMD results. RMS, root-mean-square.

We wanted to establish how different sifting methods reconstruct waveform shape in the presence of noise (Fig. 3). Ten seconds of a 4 Hz nonsinusoidal iterated sine signal with white noise was simulated. The standard deviation of zero-mean white noise (root-mean-square of noise, RMS noise) was varied as the shape of the wave remained constant with 8th-order iterated sine (frequency distortion FD = 68%, see materials and methods). Iterated masking was compared with existing dyadic masking and ensemble sifting techniques.

First, we measured performance by computing Pearson correlation of reconstructed and ground truth waveform shape measured by the instantaneous frequency (Fig. 3*A*).

For low-to-medium noise amplitudes (*σ*_noise_ ⪅ 1 with normalized signal amplitude of one), existing techniques were sufficient to represent the waveform shape well. Ensemble-sift reconstructed waveform shape had a high correlation with the ground truth shape with *r* > 0.75, but its performance quickly deteriorated past *σ*_noise_ = 0.1. Dyadic mask sift had poor shape reconstruction for no noise but performed well from *σ*_noise_ = 0.1 onward. The novel iterated masking performed well except for a dip in performance around ultra-low noise below *σ*_noise_ = 0.1. This was found to be the level where noise RMS is equal to the amplitude of one of the higher signal harmonics. As such, this harmonic was sometimes included in the IMF of interest and sometimes not, depending on exact noise details. This introduced stochastic mode mixing (see also Supplemental material and Supplemental Fig. S1). Noise levels in neurophysiological data are seldom this low. However, we were able to automatically identify these pathological cases because the mask did not reach a stable equilibrium and the maximum number of iterations was reached (red shading).

For high noise amplitudes (1 ⪅ *σ*_noise_ ⪅ 2), the new itEMD significantly outperformed the existing techniques. Ensemble sift produced sine waves and failed to capture nonsinusoidal waveform behavior (correlation near zero). Dyadic mask sift suffered from mode mixing, with the waveform split across IMF-4 and IMF-5 (Fig. 1*D*). Because of this, dyadic masking failed to accurately reconstruct waveform shape, especially above *σ*_noise_ = 1. In contrast, itEMD accurately isolated the signal even at high noise levels (Fig. 3*B*). Its reconstructed shape correlated with the ground truth significantly better than the existing techniques with *P* < 0.01 (Bonferroni corrected across 100 noise levels) across the high noise range 1 _⪅_ *σ*_noise_ ⪅ 2.

In the region of very high noise with *σ*_noise_ > 2, all methods behaved as dyadic filters and failed to capture waveform shape. This is because higher Fourier harmonics encoding shape details became submerged in noise, making it impossible to recover the nonsinusoidal shape.

We also evaluated mode mixing performance by computing the PMSI (pseudo-mode splitting index, Fig. 3*C*), a mode mixing metric previously used in the literature (22). A high PMSI value indicates severe mode mixing and poor sift.

For low noise amplitudes (*σ*_noise_ ⪅ 0.3), the dyadic mask sift produced the least amount of mode mixing (lowest PMSI). In this region, itEMD was again susceptible to stochastic mode mixing due to noise levels matching higher harmonics, increasing the PMSI. Ensemble sift had the most mode mixing in this region.

For medium-to-high noise (0.3 ⪅ *σ*_noise_ ⪅ 2), itEMD had significantly less mode mixing than existing techniques (lowest PMSI *P* < 0.01, Bonferroni-corrected). Ensemble sift had a largely unchanging amount of mode mixing, suggesting it was driven by the added noise. Dyadic masking had the most mode mixing in this region.

All three methods had similar PMSI in the very high noise region with *σ*_noise_ > 2 due to inherent dyadic filtering behavior of EMD.

Neurophysiological signals typically show auto-correlated 1/*f* noise (also termed aperiodic activity or fractal noise) (47). To verify our technique works with 1/*f* noise simulations, we re-ran all the main analyses with brown noise (Supplemental material S3). As with white noise, itEMD outperformed existing techniques over a wide range of parameters.

Finally, we compared mask frequency stability across itEMD iterations for a mode known to have signal (IMF-4) and a pure noise mode (IMF-5). The mask frequency was significantly less variable when signal was present (Supplemental material S5 and Supplemental Fig. S5).

#### Influence of frequency distortion (nonsinusoidality).

Highly nonsinusoidal waveforms have been observed across a variety of neural data (see introduction). As such, we compared existing techniques and itEMD performance in data with progressively more waveform distortion. Ten seconds of a 4 Hz nonsinusoidal iterated sine signal with white noise of standard deviation *σ*_noise_ = 1 was simulated. Frequency distortion was varied by iterating the sine function between 1 and 18 times. Each frequency distortion level was simulated with *n* = 100 different noise realizations. Performance was again compared using Pearson correlation to ground truth shape and the PMSI.

**Figure 4.**
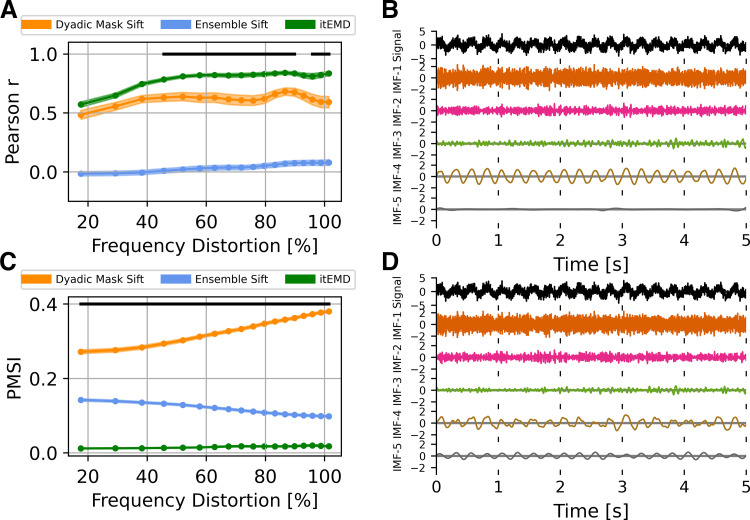
Influence of frequency distortion on empirical mode decomposition (EMD) performance in simulated data. *A*: Pearson correlation coefficient between reconstructed and ground truth instantaneous frequency against increasing frequency distortion. *B*: example 5 s of itEMD sift results for *σ*_noise_ = 1, frequency distortion (FD) = 80%. *C*: pseudo-mode splitting index (PMSI) against frequency distortion, with higher PMSI values indicating higher mode mixing. Means ± standard error across *n* = 100 noise realizations (shaded) shown. Black line indicates regions where itEMD performs significantly better than the best of the other techniques with *P* < 0.01 (multiple comparisons Bonferroni corrected). The novel iterated masking EMD (itEMD) performs significantly better for highly nonsinusoidal data in the region FD > 50% with reduced mode mixing and accurate waveshape reconstruction. *D*: example 5 s of dyadic mask sift results for *σ*_noise_ = 1, FD = 80%. Intrinsic mode function (IMF)-4 shows significantly more mode mixing with existing methods than with our novel itEMD.

Iterated masking performed significantly better than the existing methods for highly nonsinusoidal signals (Fig. 4, Bonferroni-corrected *P* < 0.01 for lowest PMSI and higher Pearson *r*). Dyadic mask shape correlation with ground truth was not significantly different from itEMD for FD < 50%, but severe mode mixing was present. This meant the average frequency and amplitude were poorly reconstructed. At this noise level, ensemble sift behaved as a dyadic filter and completely failed to capture waveform shape. It produced a sinusoid at the dyadic boundary of *f* = 4 Hz with no nonsinusoidality. itEMD performance also improved with increasing frequency distortion. This is due to higher frequency harmonics increasing in magnitude with more shape distortion, allowing better convergence of itEMD.

#### Reconstructed waveform.

**Figure 5.**
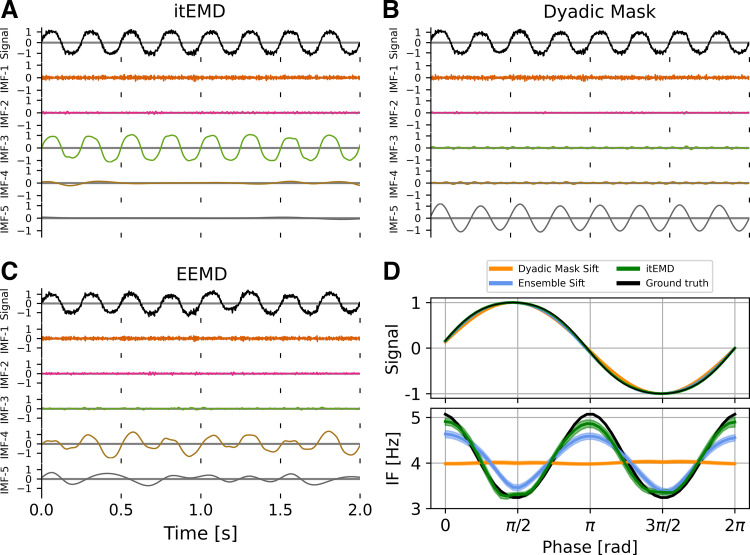
Example 2 s of sifting results for 4th-order iterated sine with white noise *σ*_noise_ = 0.1 in simulated data. *A*: intrinsic mode functions (IMFs) for the novel iterated masking empirical mode decomposition (itEMD). Iterated sine is in IMF-3 with very little mode mixing. *B*: IMFs for dyadic mask sifting. Signal is split between IMF-3 and IMF-5. C: IMFs for ensemble sifting. Iterated sine is mostly in IMF-4, but mode mixing is present. *D*, *top*: average reconstructed waveform, *bottom*: reconstructed phase-aligned instantaneous frequency (IF); means (line) ± standard error across cycles (shaded) shown. Dyadic mask sift waveform (orange) fails to reconstruct nonsinusoidality. Ensemble sift recovers more shape detail but suffers from high mode mixing. itEMD is able to reconstruct more of the waveform shape than either existing method while lowering mode mixing. EEMD, ensemble empirical mode decomposition.

Next, we looked at individual IMFs and reconstructed waveforms and their instantaneous frequency (Fig. 5). As expected from the Pearson *r* and PMSI results in Fig. 4, itEMD best reconstructed a highly nonsinusoidal waveform in the presence of noise. A noise level of *σ*_noise_ = 0.1 and 4th-order iterated sine were chosen as they are qualitatively similar to experimental LFP and MEG recordings analyzed (cross-reference Fig. 7). The ensemble sift was able to capture most of the nonsinusoidality but suffered from heavy mode mixing (PMSI = 0.0943). The dyadic mask sift had slightly less mode mixing (PMSI = 0.0923) but failed to capture any nonsinusoidal waveform shape details. The novel iterated masking captured the waveform shape best with the least mode mixing (PMSI = 0.0003). However, the waveform was still not perfectly reconstructed. This was due to *1*) some of the harmonics encoding the finer details being lower in spectral density than the noise and *2*) due to intrinsic finite bandwidth of EMD modes (see Supplemental material and Supplemental Fig. S2). However, itEMD performed significantly better than the other techniques with the root-mean-square error to the ground truth instantaneous frequency being significantly lower (*P* = 1.75 × 10^−9^ vs. dyadic mask, *P* = 0.045 vs. ensemble sift, Bonferroni-corrected).

#### Influence of signal sparsity.

**Figure 6.**
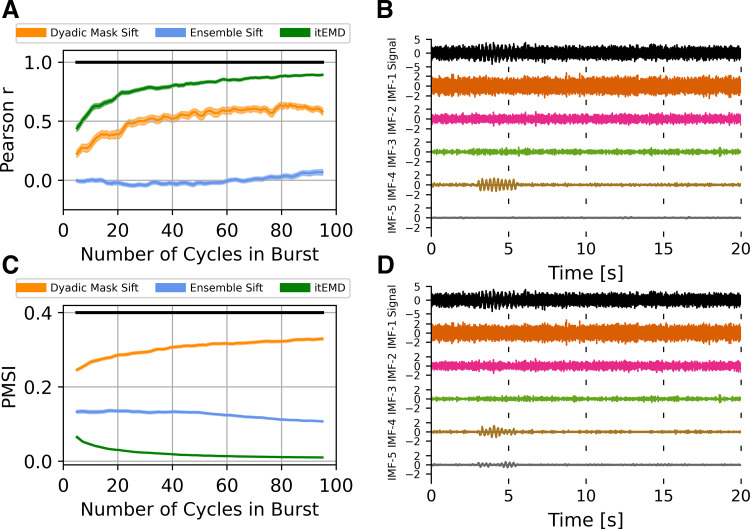
Influence of signal sparsity on empirical mode decomposition (EMD) performance on simulated data. *A*: Pearson correlation coefficient between reconstructed and ground truth instantaneous frequency against increasing burst length. *B*: example iterated masking EMD (itEMD) sift results for *σ*_noise_ = 1, FD = 68%, 10 cycles. *C*: pseudo-mode splitting index (PMSI) against burst length. Means ± standard error across *n* = 100 noise realizations (shaded) shown. Black line indicates regions where itEMD performs significantly better than the best existing technique with *P* < 0.01 (multiple comparisons Bonferroni corrected). The novel itEMD performs significantly better for a wide range of burst durations. *D*: example dyadic mask sift results for *σ*_noise_ = 1, FD = 60%, 10 cycles in burst. Iterated sine function is captured by intrinsic mode function (IMF)-4 and IMF-5 due to mode mixing. FD, frequency distortion.

Neural activity often consists of intermittent bursts (17). To test itEMD performance when signal is sparse, we simulated 25 s of zero-mean white noise with *σ*_noise_ = 1, to which we added a 4 Hz nonsinusoidal 8th-order iterated sine signal with frequency distortion FD = 68% and variable length of 5–100 cycles. When reconstructing waveform shape of this burst, itEMD performed significantly better than either the dyadic mask sift, or the ensemble sift (Fig. 6). Even in the presence of high noise and nonsinusoidality, itEMD was able to extract the burst and identify its waveform shape. Its correlation with ground truth waveform shape was significantly higher than the other methods for all burst lengths considered (Fig. 6*A*, *P* < 0.01, Bonferroni corrected across number of cycles in the burst). Mode mixing measured by the PMSI was also significantly lower than with the existing methods (Fig. 6*C*, *P* < 0.01, Bonferroni corrected). Performance of itEMD improved as burst length increased. Overall, this demonstrates the potential benefits of itEMD when characterizing transient bursts, which is increasingly used to describe oscillations in electrophysiological data (5).

### Application to Experimental Data

#### Rat local field potential.

**Figure 7.**
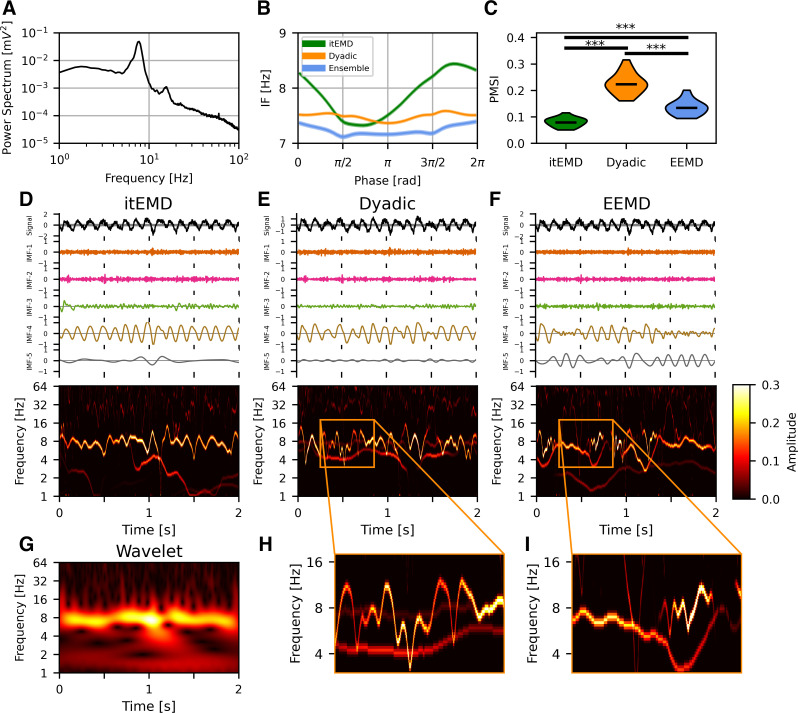
Rat hippocampal local field potential (LFP) results. *A*: power spectrum of the full recording showing a θ peak harmonic. *B*: phase-aligned instantaneous frequency of θ cycles (means ± standard error across all cycles shown). Existing methods including dyadic mask sift and ensemble sift fail to capture high nonsinusoidality of θ oscillations unlike iterated masking empirical mode decomposition (itEMD). *C*: violin plots of the pseudo-mode splitting index (PMSI, a measure of mode mixing) across *n* = 20 segments of the 1,000 s recording. Iterated masking had significantly lower PMSI than both dyadic mask sift and ensemble sift (*P* < 10^−6^, Bonferroni-corrected across methods). *D*, *top*: example itEMD sift results from 2 s of the LFP recording, *bottom*: Hilbert–Huang Transform (HHT) for the same data. θ Oscillations are well-captured by intrinsic mode function (IMF)-4 with minimal mode mixing. *E* and *F*: same as *D* but for the dyadic mask sift and ensemble EMD (EEMD). Significant mode mixing is present. *G*: wavelet transform of the same data as in *D*–*F*. Similar dynamics to the HHT in *D* are present but with lower resolution. Compared with *E* and *F*, we see the artifacts in poor sifts (red arrows). *H* and *I*: expanded sections showing mode mixing. ***P < 0.001 (Bonferroni-corrected).

We first validated our technique by applying it to the well-understood hippocampal θ signal in a 1,000 s recording of publicly available rat hippocampal LFP data. The recording was split into *n* = 20 segments of 50 s each. This θ oscillation has been previously observed to be nonsinusoidal with, on average, a faster ascending than descending edge (8, 48, 49). Our novel iterated masking EMD (itEMD) converged after *n*_iter_ = 6 ± 1 iterations and extracted cycles with a wide instantaneous frequency sweep (Fig. 7). It reproduced the known shape with a faster leading edge (leading edge frequency 7.87 ± 0.02 Hz, falling edge frequency 7.62 ± 0.02 Hz, means ± SE, *P* = 5.9 × 10^−21^ on a paired *t* test across all cycles). In comparison to itEMD, existing ensemble and dyadic mask sifting failed to capture the high nonsinusoidality of this oscillation. Existing methods also suffered from higher mode mixing as measured by the PMSI (lowest PMSI for itEMD with Bonferroni-corrected *P* < 10^−6^). This was confirmed by visualizing the Hilbert–Huang transforms, where θ IMF has the cleanest sweep for itEMD. This could allow for improved cross-frequency coupling analysis.

To compare this analysis with more traditional methods, we also computed the wavelet transform (Fig. 7*G*). The spectrum was qualitatively very similar to the itEMD HHT except with a smoothed lower time-frequency resolution. The wavelet transform also confirmed the artifactual components present in HHTs from existing methods. This was as there was neither continuous low-frequency component nor high/low frequency switching seen in dyadic masking and EEMD, respectively, due to mode mixing.

#### Human magnetoencephalography.

**Figure 8.**
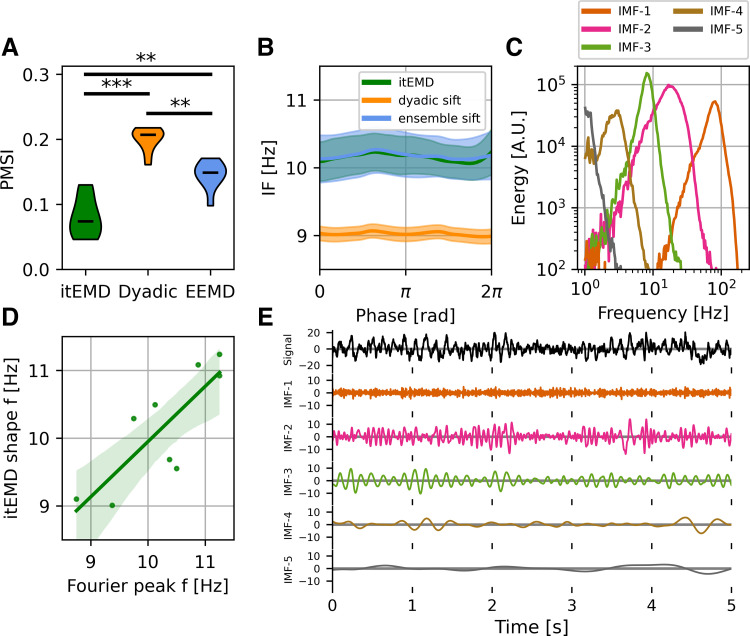
Human magnetoencephalography (MEG) occipital α results. *A*: group-level violin plots of the pseudo-mode splitting index (PMSI, a measure of mode mixing). Iterated masking had significantly lower PMSI than both dyadic mask sift (*P* = 6*e*−5), and ensemble sift (*P* = 0.0062, both Bonferroni-corrected). *B*: group-level phase-aligned instantaneous frequency (means ± standard error shaded). Both iterated masking empirical mode decomposition (itEMD) and ensemble empirical mode decomposition (EEMD) detect the 10 Hz occipital α oscillation with no significant nonsinusoidality. Masked sift fails to capture the oscillation well due to mode mixing. *C*: one-dimensional (1-D) Hilbert–Huang Transform (HHT) for intrinsic mode functions (IMFs) from an example subject. *D*: mean subject phase-aligned instantaneous frequency against peak α frequency from the Fourier power spectral density. Iterated masking linearly reproduces between-subject variability in α frequency (*P* = 0.00739, *F* test against constant null hypothesis). *E*: example 5 s of raw itEMD sift results. α Oscillations are in IMF-3 with sharp features in IMF-2 and minimal mode mixing. **P < 0.01, ***P < 0.001 (Bonferroni-corrected).

For further validation, we analyzed 10 min of occipital resting-state data from each of 10 subjects (Fig. 8). One subject was excluded as their spectrum did not show an α peak. We found itEMD successfully and rapidly converged on the intermittent α oscillation around 10 Hz (*n*_iter_ = 5 ± 1 iterations across all subjects, means ± standard deviation). Compared with dyadic mask sift and ensemble sift, mode mixing measured by the PMSI was significantly lower (*P* = 6.0 × 10^−5^ vs. dyadic mask, *P* = 0.0033 vs. ensemble sift, Bonferroni-corrected paired *t* test across subjects).

α Peak frequency is known to vary between people, conditions, and changes with age (50, 51). EMD has the advantage of representing spectral components with fewer assumptions about frequency bands, unlike traditional Fourier analyses focusing on the 8–12 Hz power for example. As such, it may be well-suited to represent these inter- and intraindividual spectral differences. Variability in α peak frequency was also seen in data analyzed here. Moreover, itEMD was able to replicate this result, with the mean subject phase-aligned instantaneous frequency found to be linearly related to spectral peak frequency (Fig. 8*D*, *F* = 13.89, *P* = 0.00739). Recordings also showed the presence of β-band elements around 20 Hz in IMF-2, which were also present in the Fourier spectrograms (Fig. 8*E* and Supplemental Fig. S5C).

## DISCUSSION

In this paper, we introduced a novel way of performing empirical mode decomposition (EMD) called iterated masking EMD (itEMD). This technique is capable of robustly decomposing signals into spectral components in the presence of noisy, sparse, and highly nonsinusoidal oscillations. In itEMD, masking signals are introduced at frequencies identified by an iterative, data-driven process to solve the mode mixing observed with other EMD methods. We demonstrated the utility of this sifting technique in comparison with existing solutions to the mode mixing problem, especially in highly noisy and nonsinusoidal signals.

We validated the method using rat LFP and human MEG recordings. We found rat hippocampal θ to be highly nonsinusoidal with a faster ascending edge as previously reported (8, 48, 49). Intermittent human occipital α was found to be nearly sinusoidal with a between-subject variable peak frequency around 10 Hz. Mode mixing was found to be significantly lower when using itEMD compared with ensemble and dyadic mask sifting in these neurophysiological recordings. Iterated masking EMD has the potential to enable more widespread use of EMD in neurophysiology and shed light on single-cycle dynamics across a wide range of modalities and conditions. It automates the selection of mask frequencies and can thus enable a wide range of analyses about bursts of neural activity, genuine cross-frequency coupling, and analysis of neural phase.

We performed extensive simulations across a wide range of noise, sparsity, and nonsinusoidality (frequency distortion) parameters. In our validation of itEMD using simulated data, we used white noise. We focused on white noise because in this case results become independent of simulated signal frequency and hence ought to be applicable to any common noise structure. However, our technique is just as applicable to different noise structures (e.g., brown noise; Supplemental material S3).

The stability of the mask equilibrium found by itEMD was found to be higher for modes containing genuine signal (Supplemental Fig. S5). It was observed that the equilibrium was well-defined for IMFs displaying consistent oscillations with between-iteration mask frequency changes of less than 3% and rapid convergence (<10 iterations). This makes sense as itEMD found the equilibrium where IMF frequency matched mask frequency, as described in materials and methods. Conversely, IMFs containing mostly noise had highly variable mask frequencies changing by more than 5% between iterations with no well-defined equilibrium. This could be used to assess whether an oscillation is present in any given segment of data in addition to existing ways of doing this such as amplitude and period consistency (37).

### Comparison with Existing Analysis Methods

As itEMD is an EMD-based technique, this paper focused on direct comparisons with other EMD sifting techniques. As mentioned, a key reason why EMD has scarcely been applied to neurophysiological data is mode mixing (13, 14). When oscillations of interest are present across multiple IMFs, their interpretation and further analysis is made much more difficult. Iterated masking EMD significantly reduced mode mixing [measured by the PMSI as per previous literature before (22)] whilst still being able to reconstruct nonsinusoidal oscillations in the signal. Compared with the existing masked sift, itEMD has the advantage of being fully data-driven and avoiding manual mask optimization.

Non-EMD based analysis methods may complement itEMD. Traditionally, analysis has been done by calculating the Hilbert transform on narrowband filtered data (8). This works well if frequencies of interest are defined a priori. However, it poses limitations on how nonsinusoidal oscillations can be and does not allow for large between-subject variabilities. Furthermore, the use of Fourier filters may introduce bias into the analysis (10, 52). More recently, methods based on detecting phase control points (peaks, troughs, etc.) have been developed (37). These provide important summary statistics for cycles, such as peak-trough asymmetry and rise-decay asymmetry. EMD-based analysis describes the shape with phase-aligned instantaneous frequency without restricting analysis to certain phase points. The cycle-by-cycle approach could thus be cross-validated by itEMD detecting asymmetry around the phase points used for its statistics. Finally, additional novel algorithms for extracting summary waveforms for a whole recording have been developed (53, 54). Unlike itEMD and the techniques described earlier, these are however not sensitive to changes in waveform within a recording.

ItEMD also has the potential to complement the analysis of common task-related data. However, different analysis methods are needed. Traditionally, activity (or the Fourier/wavelet spectrum) is averaged across trials. Using the Hilbert–Huang transform (HHT) without smoothing, this may average to zero. There is however increasing recognition that individual bursts and cycles may give us additional information about the brain’s functions (16, 17). As such, we recommend making use of trial-specific information EMD produces, such as building a general linear model with the instantaneous frequency and amplitude associated with each cycle (see Ref. 11 for an example of such an analysis and Fig. 7, *D*–*G* to compare HHT with a wavelet transform).

### Limitations When using itEMD

Although itEMD represents a significant step toward extracting nonsinusoidal neural oscillations in a data-driven way, there may be situations where other techniques are more appropriate. Here we draw attention to a few cases where this may be the case.

Iterated masking EMD works to capture more waveform shape details by adapting the bandwidth of an IMF to include more signal from higher frequency harmonics (Supplemental material S2). However, together with capturing more shape details, this also increases the amount of noise in the IMF. If oscillations being studied are not expected to be nonsinusoidal, or such features are not of interest, other methods (such as a carefully designed manual mask for masked sift, or Fourier techniques) may be better at boosting signal-to-noise ratio. Indeed, it was found that at high noise levels, the SNR boost from itEMD is lower than other sifting methods (Supplemental Fig. S2).

As with most EMD-based algorithms, itEMD also has a fundamental limitation in how large a single IMF bandwidth can be. It has been previously shown that the original EMD algorithm differentiates between a single-amplitude-modulated tone and two separate tones based on their rates of extrema and amplitude ratios (55). This means that when shape-encoding harmonics are much higher in frequency than the base frequency (approximately *af*^2^ *>* 1 for amplitude ratio *a* and frequency ratio *f* of base to harmonic as explained in Ref. 55), itEMD will tend to treat these as two separate oscillations. If they need to be treated as a single oscillation, researchers should use masks specifically designed for overcoming this limit (27) or non-EMD based waveform analysis techniques [e.g., cycle-by-cycle analysis (37)]. It is still a matter of debate as to when higher-frequency signals constitute harmonics as opposed to genuine separate oscillations (56, 57). This issue is important, as harmonics can cause spurious cross-frequency coupling if not accounted for properly, and ongoing research in our group is attempting to clarify this issue. Finally, in high levels of noise, high-frequency harmonics may be below the noise level (Supplemental material S1). When this happens, itEMD will only partially reconstruct the waveform shape. This is because EMD works locally by finding extrema, and if they are dominated by noise on a given scale, EMD will not be able to identify signal. Our technique shares this limitation with all existing EMD-based analysis methods. However, even at high noise levels when this happens, our technique still had significantly lower mode mixing and preserved the correlation between ground truth and reconstructed instantaneous frequency.

In this paper, we measured mode mixing with the previously used pseudo-mode splitting index (PMSI) (22). This metric assumes the sift works optimally when IMFs are orthogonal. As we are attempting to find a basis of components for the data, orthogonality is desirable (see e.g., section 6 in Ref. 10). However, this is not always guaranteed if strong harmonic or coupled components are present in the data. Correlation between modes should be assessed for each application and sift parameters adjusted if excessive correlation is found.itEMD was designed to handle sparse oscillations, but it may be necessary to adjust the amplitude weighting method if signals of interest are very sparse (<10% of a data segment being processed). This can be done by changing the weighting of instantaneous frequency when iterating. Here, we used weighting by the square of instantaneous amplitude (*IA*^2^, instantaneous power) at each iteration. Higher powers of instantaneous amplitude may help if sparsity is preventing itEMD from converging on oscillations of interest. Conversely, weighting by lower powers may be appropriate if minute IA fluctuations are driving spurious results.

In this work, we defined iteration convergence when the relative mask change between iterations was under 10%. We also tried continuing for ten iterations after this point and averaging the resulting IMFs to verify the robustness of our threshold. It was qualitatively observed that only minimal changes occurred after the 10% convergence point. However, in datasets not studied here, it may be necessary to tune the convergence criterion or take the average of a few iterations after soft convergence around 10% depending on exact noise structure present. Our analyses were all also relatively insensitive to the choice of the maximum number of iterations *n*_max_ as itEMD converged rapidly in most cases. However, we cannot guarantee this in all possible applications. *n*_max_ should be high enough so the stopping criterion is reached in most cases. The current implementation raises a warning if the maximum number of iterations has been reached and the plausibility of the decomposition should be checked, for instance by way of comparison with existing EMD methods.

When analyzing our MEG data, we segmented the recordings into 10 parts of about a minute each. itEMD implicitly assumes that the mean frequency of oscillations of interest is not changing greatly and an equilibrium can be found. Hence, to allow for drifting in the spectral peak frequency segmented data were used. However, it was found α frequency did not vary significantly during this resting-state experiment and applying itEMD on full recordings yielded very similar results (see Supplemental material S5). In general, we recommend segmenting the data if peak frequencies may shift over time (e.g., in drug induction or task data). Furthermore, here we analyzed a single MEG sensor. This means that the recorded signal may be a superposition of multiple underlying sources. To separate sources both spectrally and spatially, other methods should be used in conjunction with itEMD, for instance independent component analysis (ICA) or source reconstruction.

In summary, we have introduced a novel way to robustly extract oscillatory modes from neural recordings using iterated masking EMD. Our method has all the advantages of using EMD while resolving limitations of existing sifting techniques by significantly reducing the mode mixing and robustly capturing oscillations even in the presence of noise, sparsity, and high nonsinusoidality. By validating it on extensive simulations and real multimodal, multispecies data, we have demonstrated its potential to bring the full power of EMD into neurophysiology and help elucidate the role of dynamic neural oscillations in behavior and disease.

## SUPPLEMENTAL DATA

10.6084/m9.figshare.16680901.v1 Supplemental material and Supplemental Figs. S1, S2, and S5: https://doi.org/10.6084/m9.figshare.16680901.v1.

## GRANTS

This research was funded in part by the Wellcome Trust Grant No. 203139/Z/16/Z. Research was also supported by the NIHR Oxford Health Biomedical Research Centre, the Wellcome Trust (106183/Z/14/Z, 215573/Z/19/Z), the New Therapeutics in Alzheimer’s Diseases (NTAD) study supported by UK MRC and the Dementia Platform UK (RG94383/RG89702), an EU European Training Network grant (euSSN; 860563), and the MRC Development Pathway Funding Scheme (award reference MR/R006423/1).

## DISCLOSURES

No conflicts of interest, financial or otherwise, are declared by the authors.

## AUTHOR CONTRIBUTIONS

M.S.F. conceived and designed research; M.S.F. performed experiments; M.S.F. analyzed data; M.S.F., A.J.Q., C.E.W., and M.W.W. interpreted results of experiments; M.S.F. prepared figures; M.S.F. drafted manuscript; M.S.F., A.J.Q., C.E.W., and M.W.W. edited and revised manuscript; M.S.F., A.J.Q., C.E.W., and M.W.W. approved final version of manuscript.

## ENDNOTE

At the request of the authors, readers are herein alerted to the fact that additional materials related to this manuscript may be found at https://gitlab.com/marcoFabus/fabus2021_itemd, https://crcns.org/data-sets/hc/hc-3, and https://camcan-archive.mrc-cbu.cam.ac.uk/dataaccess/. These materials are not a part of this manuscript and have not undergone peer review by the American Physiological Society (APS). APS and the journal editors take no responsibility for these materials, for the website address, or for any links to or from it.
